# MSCSO: A Modified Sand Cat Swarm Algorithm for 3D UAV Path Planning in Complex Environments with Multiple Threats

**DOI:** 10.3390/s25092730

**Published:** 2025-04-25

**Authors:** Zhengsheng Zhan, Dangyue Lai, Canjian Huang, Zhixiang Zhang, Yongle Deng, Jian Yang

**Affiliations:** School of Automation Science and Engineering, South China University of Technology, Guangzhou 510641, China

**Keywords:** UAV path planning, sand cat swarm optimization, chaotic mapping, Lévy flight long-step perturbation, nonlinear particle swarm optimization weight, elite mutation mechanism

## Abstract

To improve the global search efficiency and dynamic adaptability of the Sand Cat Swarm Optimization (SCSO) algorithm for UAV path planning in complex 3D environments, this study proposes a Modified Sand Cat Swarm Optimization (MSCSO) algorithm by integrating chaotic mapping initialization, Lévy flight–Metropolis hybrid exploration mechanisms, simulated annealing–particle swarm hybrid exploitation strategies, and elite mutation techniques. These strategies not only significantly enhance the convergence speed while ensuring algorithmic precision but also provide effective avenues for enhancing the performance of SCSO. We successfully apply these modifications to UAV path planning scenarios in complex environments. Experimental results on 18 benchmark functions demonstrate the enhanced convergence speed and stability of MSCSO. The proposed method has a superior performance in multimodal optimization tasks. The performance of MSCSO in eight complex scenarios that derived from real-world terrain data by comparing MSCSO with three state-of-the-art algorithms, MSCSO generates shorter average path lengths, reduces collision risks by 21–35%, and achieves higher computational efficiency. Its robustness in obstacle-dense and multi-waypoint environments confirms its practicality in engineering contexts. Overall, MSCSO demonstrates substantial potential in low-altitude resource exploration and emergency rescue operations. These innovative strategies offer theoretical and technical foundations for autonomous decision-making in intelligent unmanned systems.

## 1. Introduction

The year 2024 signifies the emergence of the low-altitude economy, representing a landmark advancement in aerial technology. With breakthroughs in unmanned aerial vehicle (UAV) technology, various drones have been widely applied to logistics [1], exploration [2], search and rescue [3], agricultural planning [4], and other missions. Comprehensive research [5] demonstrates that the UAV industry has achieved remarkable progress over the past decade through multi-technology integration enabling cross-sector collaborative development. In security-monitoring applications, UAVs demonstrate intelligent surveillance capabilities in large-scale environments such as forested areas [6]. This demonstrates the critical role of UAVs in addressing three-dimensional (3D) path planning challenges.

The core of unmanned monitoring lies in UAV path planning, which is fundamentally an optimization problem requiring the identification of optimal solutions within specified parameter constraints to generate safe and minimal-length paths. However, this faces challenges such as ensuring detection accuracy and dynamic obstacle avoidance, especially in complex environments where algorithmic precision becomes critical. Conventional path planning methods frequently demonstrate suboptimal performance in complex 3D environments, whereas swarm intelligence optimization algorithms [7] demonstrate substantial potential with higher success rates in obstacle-dense scenarios. Their effectiveness in addressing UAV path planning [8] has been validated through numerous achievements.

Swarm intelligence optimization algorithms [9] are bio-inspired computational techniques that mimic natural biological systems’ behaviors and functionalities to optimize real-world applications. In recent years, scholars have proposed various bio-inspired algorithms based on animal behaviors, including Ant Colony Optimization (ACO) [10], Pigeon-Inspired Optimization (PIO) [11], the Nutcracker Optimization Algorithm (NOA) [12], Spider Wasp Optimization (SWO) [13], and the Genetic Algorithm (GA) [14].

The Sand Cat Swarm Optimization (SCSO) algorithm [15], inspired by the predation strategies of Felis margarita (sand cats), emulates their foraging behavior patterns, social interaction dynamics, and environmental adaptation mechanisms for solving optimization problems. SCSO combines global and local search capabilities, demonstrating rapid convergence and strong robustness. This algorithm has been successfully applied to various engineering challenges [16], including the optimization of SVM parameters for Alzheimer’s diagnosis [17], the enhancement of wireless sensor network coverage [18], and the construction of rockburst damage assessment models in mines [19], thereby demonstrating its competitiveness.

When applying swarm intelligence algorithms to UAV path planning, each method presents unique strengths and limitations [20]. For instance, Genetic Algorithms (GAs) demonstrate strong global search capabilities but suffer from slow convergence rates. The particle swarm optimization (PSO) algorithm features straightforward implementation and rapid initial convergence, but suffers from limited local search precision in high-resolution scenarios. Ant Colony Optimization (ACO) shows remarkable robustness in path discovery but relies heavily on empirically tuned parameters. While the SCSO algorithm maintains a balance between exploration and exploitation dynamics, it demonstrates a longer computation time in global search phases when handling high-complexity problems. Recent advancements have predominantly focused on algorithmic hybridization or multi-strategy enhancements, exemplified by Wang et al. [21]’s cluster-based multi-space cooperative PSO (utilizing parallel search subspaces), Jia et al. [22]’s noise-enhanced SCSO with Gaussian perturbations, X. Li et al.’s multi-strategy SCSO [23], Adegboye et al. [24]’s dynamically improved SCSO with fuzzy logic-based adaptive parameters, Kiani et al. [25]’s chaotic SCSO with Tent mapping-based initialization, and Y. Li et al. [26]’s elite-guided SCSO with stochastic variation operators. While recent studies have advanced SCSO, it remains unexplored for UAV 3D path planning. This work pioneers the adaptation of SCSO to this domain, addressing gaps in predator-inspired optimization for complex 3D environments. Building upon these developments, this study proposes a Modified Sand Cat Swarm Optimization (MSCSO) specifically designed for UAV path planning in complex 3D environments.

To improve the global search efficiency and dynamic adaptability of SCSO, MSCSO is proposed by integrating four innovative strategies. During the initialization phase, chaotic mapping is integrated with opposition-based learning to generate population vectors that uniformly span the solution space, thereby avoiding falling into local optima. In the global exploration stage, Lévy flight operators are combined with the Metropolis to achieve global fast exploration. In the later exploitation phase, an innovative hybrid probability adaptive mechanism is proposed. This mechanism combines two components. One is the dynamic perturbation strategy from the simulated annealing algorithm and the other is the improved PSO social cognitive model. The improved PSO model specifically uses nonlinear inertia weights. The mechanism adjusts the strategy selection probability in real time. This adjustment is based on population diversity indicators. Subsequently, an elitism preservation strategy dynamically replaces suboptimal candidate solutions via Gaussian mutation. These strategies collectively enable MSCSO to attain a superior balance between exploratory breadth and exploitative depth in complex UAV path planning tasks. The primary contributions of this research are threefold:

(1) Development of MSCSO through four strategies. These strategies significantly boost the algorithm’s convergence speed while maintaining accuracy.
(2) The performance of MSCSO is evaluated using 18 classical benchmark functions and compared against seven renowned algorithms to demonstrate its effectiveness.
(3) We further confirm the superiority and robustness of MSCSO in practical applications by employing it in eight diverse UAV path planning scenarios, each varying in complexity.

The paper is organized as follows: Section 2 provides a detailed description of UAV kinematic constraints and cost functions. Section 3 reviews the original SCSO algorithm and introduces the MSCSO technique. Section 4 presents experimental validation. Section 5 presents a summary and the future prospects of this research.

## 2. Kinematic Analysis and Cost Function

This study formulates the UAV path planning problem as an optimization framework, where a comprehensive cost function is developed to simultaneously address multiple optimization objectives while satisfying operational constraints specific to unmanned aerial vehicles. The mathematical formulation integrates both performance metrics and physical limitations. A detailed description is provided as follows [27].

### 2.1. Kinematic Constraints

In real-world environments, the propulsion systems and maneuverability of UAVs frequently fall short in executing extreme maneuvers such as high-angle turns, rapid vertical ascents or descents, or abrupt braking. Engaging in such maneuvers substantially increases the risk of mechanical failure and performance degradation. When operating in pedestrian-dense areas, stricter limitations must be imposed on critical flight parameters including altitude and velocity, thereby mitigating the occurrence of flight accidents. To address these physical constraints, our modeling approach introduces essential simplifications. Specifically, we implement operational restrictions governing UAV flight conditions.

#### 2.1.1. Simplifications in UAV Modeling

The physical modeling of UAVs in real-world scenarios involves significant complexity due to heterogeneous geometries, localized rotational dynamics, and multifaceted influencing factors. To simplify the calculation while preserving the basic motion characteristics, we ignore secondary influences and adopt the following assumptions:

1. Macro-environmental factors: The Earth’s rotational effects and gravitational acceleration are considered invariant to UAV dynamics.
2. Local environmental factors: Atmospheric parameters including humidity, acoustic noise, and wind direction are assumed negligible, with extreme weather conditions (e.g., storms, hurricanes) excluded.
3. UAV body simplification: The UAV is modeled as a symmetric rigid body with uniform mass distribution, invariant to temporal or environmental variations.
4. Trajectory: Only the translational motion of the center of mass is tracked, with all rotational dynamics referenced to this centroid.

#### 2.1.2. Dynamical Constraints of UAVs

Due to environmental constraints and UAV propulsion limitations, high-precision obstacle avoidance and data acquisition cannot be reliably achieved during high-speed flight. Consequently, this study imposes the following restrictions on UAV dynamical parameters:

1. Maximum operational speed

The flight speed constraints of UAVs are determined by their operational environments and propulsion systems specific to their models. To ensure sufficient time for obstacle-avoidance maneuvers, restrictions need to be within feasible and safe thresholds. Assuming $v_{max}$ as the maximum operational speed, the velocity during flight adheres to the limitation expressed in Equation (1):

(1) $$\left\{\begin{array}{c} v_{i}=\sqrt{v_{xi}^{2}+v_{yi}^{2}+v_{zi}^{2}}\\ v_{i}\leq v_{max} \end{array},\;i=1,2,\ldots \right.$$

The velocity components of the UAV along the axes at the i-th time instant are denoted as $\left(v_{xi}{,v}_{yi},v_{zi}\right),$ and the maximum operational speed is constrained by $v_{max}=18\;km/h$.

2. Maximum acceleration and deceleration

To ensure flight stability while preventing engine overload and avoiding attitude instability caused by excessive acceleration, the maximum acceleration/deceleration values must be strictly confined within safe operational boundaries, with specific constraints rigorously enforced according to Equation (2):

(2) $$a_{-}\leq {a_{i}\leq a}_{+},\;i=1,2,\ldots$$

The acceleration at the i-th time instant is denoted as $a_{i}$, and the maximum acceleration and deceleration are constrained by $a_{+}=1\;m/s^{2}$ and $a_{-}=-4\;m/s^{2}$.

3. Maximum yaw angle, climb angle, and dive angle

During UAV cruise operations, periodic environmental scanning and analysis are required, where complex environmental factors may pose navigational threats. To ensure flight safety, angular constraints must be imposed across all degrees of freedom. Assuming the maximum yaw angle, climb angle, and dive angle are $\alpha_{max}$, $\beta_{max}$, and $\gamma_{max}$, respectively, the angular limitations during flight are governed by Equation (3):

(3) $$\left\{\begin{array}{c} -\alpha_{max}\leq \theta_{1i}\leq \alpha_{max}\\ \gamma_{max}\leq \theta_{2i}\leq \beta_{max} \end{array},\;i=1,2,\ldots \right.$$

where $\theta_{1i}$ and $\theta_{2i}$ denote the yaw angle and pitch angle at the i-th time instant, with all maximum angular thresholds set to 45°.

4. Maximum yaw rate constraint

During high-speed turning maneuvers, UAVs may experience excessive roll or yaw angles that exceed control system response thresholds, potentially leading to a loss of stability or structural failure. Furthermore, extreme angular displacements impose critical mechanical stress on airframe integrity and induce destabilizing aerodynamic vortices, thereby escalating flight instability risks. Assuming $\omega_{max}$ denotes the maximum permissible yaw rate, the angular velocity constraints during flight are governed by Equation (4):

(4) $${-\omega }_{max}\leq \omega_{i}\leq \omega_{max},\;i=0,1,\ldots$$

where $\omega_{i}$ represents the yaw rate at the i-th time instant, with $\omega_{i}$ set to 60°/s.

5. Minimum path segment length

To maintain UAV performance and ensure operational integrity during prolonged maneuvers (e.g., sustained turns or climbs), a minimum straight-flight segment length $l_{min}$ is enforced. This constraint guarantees adequate stabilization intervals for control command execution. The limitation is defined in Equation (5):

(5) $$||\vec{P_{mn}^{\prime }P_{m,n+1}^{\prime }}||\geq l_{min}$$

where $\left||\vec{P_{mn}^{\prime }P_{m,n+1}^{\prime }}|\right|$ denotes the 3D Euclidean distance between two nodes, with $l_{min}$ configured as 5 m.

### 2.2. Cost Function

#### 2.2.1. Path Length Cost

During UAV navigation, path length constitutes one of the most critical factors affecting energy consumption. To enhance mission execution efficiency, the resultant path must be minimized. The path length cost $F_{1}$ function is quantified through Equation (6):

(6) $$F_{1}=\sum_{i=1}^{n}\left||\vec{P_{mn}^{\prime }P_{m,n+1}^{\prime }}|\right|$$

#### 2.2.2. Collision Risk Cost

**Figure 1:**
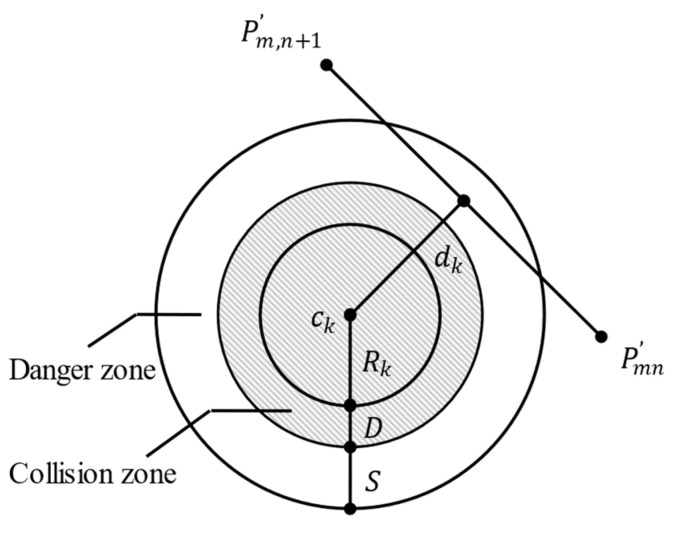
Threat cost visualization.

In addition to minimizing path length, the algorithm must guide UAVs through threat zones (i.e., obstacles in complex environments). To ensure operational safety, all threats are modeled as cylindrical obstacles, with numerous obstacles existing in the mission space. For a given path $\vec{P_{mn}^{\prime }P_{m,n+1}^{\prime }}$, the collision risk cost $F_{2,k}$ between the UAV and the k-th obstacle centered at $C_{k}$ is defined by Equation (7):

(7) $$F_{2,k}=\left\{\begin{array}{c} 0,\;\;d_{k}>S+D+R_{k}\\ S+D+R_{k}-d_{k}\;,\;D+R_{k}{\leq d}_{k}\leq S+D+R_{k}\\ \infty \;,\;d_{k}<D+R_{k} \end{array}\right.$$

where D is the UAV radius, S represents the collision avoidance safety distance (set to 10 m in this study), $R_{k}$ denotes the radius of the k-th obstacle, and $d_{k}$ is the Euclidean distance between the UAV and obstacle k, as demonstrated in Figure 1.

The total collision risk cost $F_{2}$ aggregates all obstacle costs as defined in Equation (8):

(8) $$F_{2}=\sum_{k=1}^{K}F_{2,k}$$

#### 2.2.3. Altitude Deviation Cost

**Figure 2:**
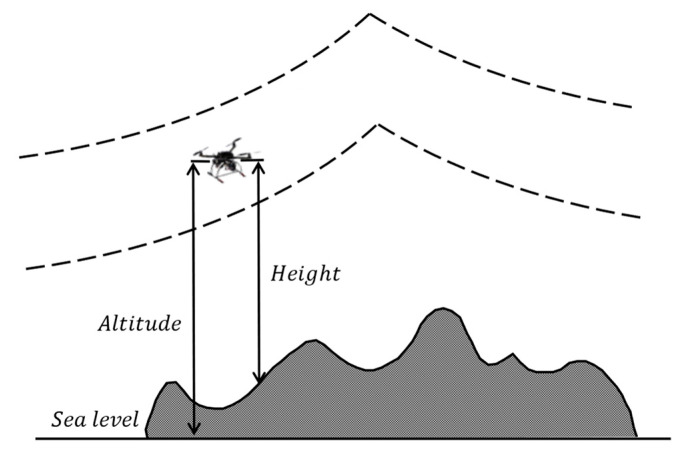
Height cost visualization.

Maintaining a controlled flight altitude is critical for UAV operations: excessive altitude leads to unnecessary energy waste, while insufficient altitude poses safety hazards to both the UAV and ground pedestrians. Assuming $h_{max}$ and $h_{min}$ denote the maximum and minimum allowable flight altitudes of the UAV, and the altitude deviation cost $F_{3}$ is defined by the piecewise function in Equation (9):

(9) $$F_{3}=\left\{\begin{array}{c} \left|h_{i}-\frac{\left(h_{min}+h_{max}\right)}{2}\right|,\;{h_{min}\leq h_{i}\leq h}_{max}\\ \infty \;\;\;\;\;\;\;\;\;\;\;,\;else \end{array}\right.$$

where $h_{i}$ represents the UAV’s relative altitude above ground at the i-th time instant, as shown in Figure 2, with $h_{max}=400\;m$ and $h_{min}=20\;m$.

#### 2.2.4. Smoothness Cost

The smoothness cost quantifies the geometric continuity of UAV trajectories by evaluating directional changes in both horizontal and vertical planes.

**Figure 3:**
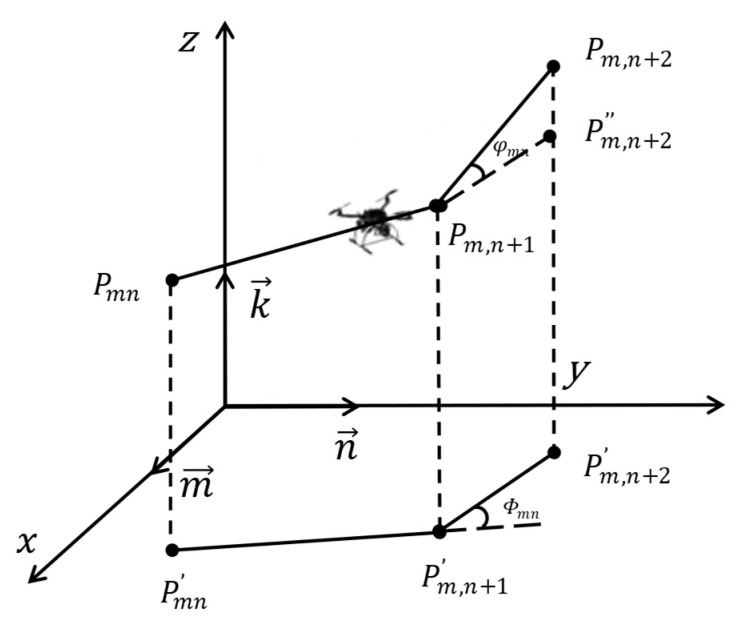
Turning and climbing angle calculation.

As shown in Figure 3, for consecutive 3D path segments $\vec{P_{mn}P_{m,n+1}}$ and $\vec{P_{m,n+1}P_{m,n+2}}$, their horizontal projections $\vec{P_{mn}^{\prime }P_{m,n+1}^{\prime }}$ and $\vec{P_{m,n+1}^{\prime }P_{m,n+2}^{\prime }}$ form the basis for angular calculations. $\varphi_{mn}$ measures the horizontal steering effort between the extended line of $\vec{P_{mn}^{\prime }P_{m,n+1}^{\prime }}$ and $\vec{P_{m,n+1}^{\prime }P_{m,n+2}^{\prime }}$, while $\phi_{mn}$ captures the vertical ascent or descent effort through the angle between $\vec{P_{m,n+1}P_{m,n+2}}$ and its horizontal projection. These angular metrics are combined through Equation (10):

(10) $$F_{4}=\varphi_{mn}+\phi_{mn}$$

#### 2.2.5. Overall Cost Function

The overall cost function F is formulated as a convex combination of individual cost components, reflecting their relative importance in path planning. These components include path length, collision risk, altitude deviation, and smoothness. The aggregated cost function is defined in Equation (11):

(11) $$F=\sum_{i=1}^{4}{b_{i}F}_{i}$$

where $b_{i}$ donates the weight for different costs.

Based on the safety priority principle and operational efficiency considerations, the weight coefficients $b_{1},{b_{2},b_{3},b}_{4}$ are defined as follows:

The highest weight $b_{2}=10$ (collision risk cost) reflects the absolute priority of flight safety.

The second-highest weight $b_{3}=8$ (altitude deviation cost) reflects that the drone must strictly avoid collisions with the ground or exceeding the maximum flight altitude.

Path length directly affects energy efficiency, but its importance is slightly lower than the safety-related costs. This means that a redundant path length can be accepted during the flight process. The third-highest weight $b_{1}=5$ balances energy consumption against safety requirements.

The angle indicator reflects the steering energy consumption and is used to optimize flight comfort. Thus, $b_{4}$ is set to 2.

## 3. SCSO and the Proposed MSCSO Method

### 3.1. Sand Cat Swarm Optimization

#### 3.1.1. Initialization

SCSO initiates the population through a uniform random distribution mechanism. The initial position of the individuals in the search space is mathematically defined as Equation (12):

(12) $$X_{i}=lb+rand\left(0,1\right)\times \left(ub-lb\right),\;i=1,2,\ldots ,N$$

where $X_{i}$ denotes the position of the i-th individual and N denotes the number of individual, while lb and ub denote the lower and upper bounds of the search space. Moreover, *rand*(0,1) denotes a uniformly distributed random variable within the range [0, 1].

#### 3.1.2. SCSO Optimization Process

1. Prey Search Phase (Exploration)

The linear attenuation formula for the sensitivity range $r_{G}$ is defined as Equation (13):

(13) $$r_{G}=2-\frac{2\times t}{T_{max}}$$

where t denotes the current iteration and $T_{max}$ denotes the maximum number of iterations. $r_{G}$ is the dynamic parameter governing the search radius.

The position update strategy is formulated as Equation (14):

(14) $$\vec{Pos}\left(t+1\right)=r\cdot \left(\vec{Pos_{bc}}-rand\left(0,1\right)\cdot \vec{Pos_{c}}\right)\;r=r_{G}\times rand\left(0,1\right)$$

where $\vec{Pos_{c}}$ represents the current individual position vector; $\vec{Pos_{bc}}$ represents the current best individual position vector; r is used in the position update strategy to control the individual’s movement step size in the search space.

2. Prey Attack Phase (Exploitation)

The circular position update formula during the prey attack phase is expressed as Equation (15):

(15) $$\vec{Pos}\left(t+1\right)=\vec{Pos_{b}}-r\cdot \vec{Pos_{rand}}\cdot cos\left(\theta \right)\;\vec{Pos_{rand}}=rand\left(0,1\right)\times \left(ub-lb\right)$$

where $\vec{Pos_{b}}$ denotes the historical best position vector; $\vec{Pos_{rand}}$ denotes the random disturbance vector; $\theta \in \left[0,\;2\pi \right]$ is the random variable for the attack angle.

3. Phase Switching Control Parameter

The position update formula during the prey attack phase is expressed as Equation (16):

(16) $$R=2r_{G}\times rand\left(0,1\right)-r_{G}$$

where R represents the phase switching control parameter, which governs the transition between exploration and exploitation phases.

The phase transition decision rule follows from Equation (17). If $\left|R\right|\leq 1$, the algorithm executes the exploration strategy (prey search phase) to enhance global search capabilities. If $\left|R\right|>1$, the algorithm implements the exploitation strategy (prey attack phase) to refine local search precision.

(17) X→t+1= Posb→t−Posrand→⋅cosθ·r→,R≤1;exploitationr→⋅Posbc→t−rand0,1⋅Posc→t,R>1;exploration

### 3.2. The Proposed Modified Sand Cat Swarm Optimization

#### 3.2.1. Population Initialization Based on Cat Mapping and Opposition-Based Learning

1. Cat Mapping

The chaotic sequence generation via the cat mapping is defined by the iterative Equation (18):

(18) $$x_{n+1}=1-mod\left(\frac{1}{x_{n}},1\right)$$

where $x_{n}$ denotes the current value of the chaotic sequence; mod represents the modulo operator; $x_{n+1}$ denotes the updated chaotic sequence value.

The chaotic sequence is mapped to the actual solution space using Equation (19).

(19) $$X_{cat}=lb+x_{n}\times (ub-lb)$$

where $X_{cat}$ represents the individual generated by the cat map chaotic initialization.

2. Opposition-Based Learning (OBL) Strategy

The opposition-based learning strategy is formulated as Equation (20):

(20) $$X_{obl}=lb+ub-X_{cat}$$

where $X_{obl}$ represents the opposition-based learning individual.

To ensure solutions remain within the search space, the constraint of Equation (21) is applied:

(21) $$X_{obl}=max(lb,min(ub,X_{obl}))$$

3. Merged Population

The merged population is constructed by combining the chaotic initialization population and the opposition-based learning population using Equation (22):

(22) $$X_{combined}=X_{cat}\cup X_{obl}=\{x_{1},x_{2},\ldots ,x_{N},{\hat{x}}_{1},\ldots ,{\hat{x}}_{N}\}$$

where $X_{combined}$ denotes the merged population.

The fitness value for each individual in $X_{combined}$ is computed as Equation (23):

(23) $$f_{i}=f\left(x_{i}\right),\forall x_{i}\in X_{combined}$$

where f denotes the fitness function, and a smaller $f_{i}$ value demonstrates a superior individual.

The optimal N individuals are selected by ranking all candidates based on their fitness values using Equation (24):

(24) $$X_{selected}=arg{min}_{X\subseteq X_{combined},\left|X\right|=N}\sum_{x\in X}f\left(x\right)$$

where $X_{selected}$ denotes the set of top-N optimal individuals selected from the merged population $X_{combined}$.

Haupt et al. mentioned that the initial population quality of swarm intelligence optimization algorithms significantly affects the global search speed and quality of the algorithm [28]. A highly diverse initial population helps to enhance the optimization effectiveness and capabilities of the algorithm.

**Figure 4:**
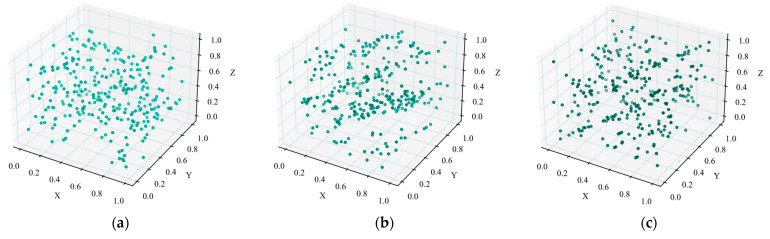
Visualization of population distributions. (**a**) Distribution of population based on random initialization. (**b**) Distribution of population based on cat mapping. (**c**) Distribution of population based on cat mapping and opposition-based learning.

Firstly, the results of the different initialization are visualized, as shown in Figure 4. To assess the quality of the randomness, the experiment is conducted in 3D space. The indicator for calculating population distribution uniformity is based on the joint variance of 3D region counts, where a smaller variance indicates a more uniform population distribution. The experiment is conducted with a population size of 300. The results show that the uniformity index of random population initialization is 105.2, the uniformity index of population initialization based on cat mapping is 49.2, and the uniformity index of population initialization based on cat mapping and opposition-based learning is 32.4. It can be demonstrated that the random population distribution is chaotic, with a high degree of dispersion and poor uniformity. The population distribution generated by simple cat mapping is more regular and uniform than a random population. The population distribution generated by combining cat mapping with opposition-based learning is the most uniform.

#### 3.2.2. Lévy Flight–Metropolis Criterion Hybrid Exploration Mechanism

1. Lévy Flight Candidate Solution Generation

To strengthen global exploration capabilities during the preliminary optimization phase, an enhanced Lévy flight mechanism is implemented. This process generates candidate solutions through heavy-tailed distribution sampling, which can be formally expressed as Equation (25):

(25) $$X_{new}=X+0.1\cdot levy\left(\beta \right)\cdot \left(ub-lb\right)\;levy\left(\beta \right)=\frac{u}{v^{1/\beta }}\;u∼N\left(0,\sigma^{2}\right),\;\;v∼N\left(0,1\right)\;\sigma ={\left(\frac{\Gamma \left(1+\beta \right)\sin\left(\pi \beta /2\right)}{\Gamma \left(\left(1+\beta \right)/2\right)\beta^{\left(1-\beta \right)/2}\sqrt{\pi /3}}\right)}^{1/\beta }$$

where $X_{new}$ represents the new population after executing the Lévy flight mechanism; $levy\left(\beta \right)$ represents the Lévy-distributed random step length and β=1.5 controls the skewness of the step length distribution, with smaller β values increasing the probability of long-range jumps; $N\left(0,1\right)$ denotes the standard Gaussian white noise with an identity covariance matrix; σ represents the normalization coefficient, ensuring the step lengths conform to Lévy distribution properties; 0.1 represents the step-size scaling factor, preventing excessive perturbations during the early search phase; and ub−lb represents the search space range, enabling adaptive scaling across all dimensions.

To ensure solutions remain within the search space, the constraint of Equation (26) is applied:

(26) $$X_{new}=max\left(lb,min\left(ub,X_{new}\right)\right)$$

2. Metropolis Criterion for Solution Acceptance

In dynamic updates, the Metropolis criterion adjusts its behavior based on the temperature phase as shown in Equation (27). In the high-temperature phase, it allows accepting new states with larger energy differences ΔE from the current state, while in the low-temperature phase, only new states with smaller energy differences are accepted.

(27) $$P_{accept}=\left\{\begin{array}{ll} 1 & \Delta E<0\\ exp\left(-\Delta E/T\right) & otherwise \end{array}\right.\;\Delta E=f\left(X_{new}\right)-f\left(X\right)\;T=T_{0}\cdot 0.95^{t}$$

where ΔE represents the fitness difference between the new and current solutions, reflecting the quality change; T represents the temperature parameter. $T_{0}$ represents the initial temperature, governing the acceptance probability in early iterations, and $T_{0}=10$; 0.95 represents the cooling coefficient that balances the temperature decay rate (5% reduction per iteration); $P_{accept}\in \left[0,\;1\right]$ is the probability of accepting a new solution. When T→0, the criterion degenerates to a greedy strategy (accepting only better solutions).

#### 3.2.3. Hybrid Exploitation Strategy

1. Simulated Annealing Perturbation

To enhance the local exploitation capability in the later exploitation phase while preventing premature convergence, this component introduces an adaptive perturbation generation mechanism based on simulated annealing. The perturbation magnitude δ dynamically decays as iterations progress with Equation (28). Then, the Metropolis criteria are used to determine whether candidate solutions are accepted. Moreover, to ensure solutions remain within the search space, the constraint of Equation (26) is applied.

(28) $$\;\;\;\;\delta =0.1\left(ub-lb\right)\left(1-t/T_{max}\right)\cdot N\left(0,1\right)\;{\;\;\;\;\;\;\;\;\;X}_{new}=X+\delta$$

2. Social Cognitive Velocity Update Model

During the development phase, an improved PSO mechanism is adopted, as shown in Equation (29):

(29) $$\left\{\begin{array}{c} V=wV+c_{1}rand(X_{global}-X)+c_{2}rand(\bar{X}-X)\\ X_{new}=X+0.1V \end{array}\right.\;w=\frac{0.9}{1+exp\left(0.1\cdot \left(t-T_{max}/2\right)\right)}\;c_{1}=2.0-1.5\left(t/T_{max}\right)\;c_{2}=1.0+1.0(t/T_{max})$$

where V describes the direction and step size of the motion and is initialized to 0. w represents the nonlinear inertia weight; $X_{global}$ denotes the current global best solution, guiding individuals toward elite regions to enhance convergence; $\bar{X}$ represents the population mean position, reflecting the central tendency of swarm distribution to maintain diversity; $c_{1}$ represents the cognitive coefficient, linearly decreasing from 2.0 to 0.5, to adjust the attraction toward $X_{global}$; $c_{2}$ represents the social coefficient, linearly increasing from 1.0 to 2.0, to adjust the attraction toward the population’s average position. To ensure solutions remain within the search space, the constraint of Equation (26) is applied.

3. Hybrid Probability Adaptation Mechanism

The probability of strategy selection $P_{hybrid}$ will be used to balance the usage ratio of the two strategies. If $rand()<P_{hybrid}$, the optimization executes the perturbation strategy; otherwise, it executes the quick update strategy, as shown in Equation (30):

(30) $$P_{hybrid}=0.5+0.2\cdot \frac{div}{max\left(div\right)}\;div=\frac{1}{N}\sum_{i=1}^{N}\left||X_{i}-\bar{X}|\right|$$

where div denotes the population diversity metric at the t-th iteration, calculated as the sum of Euclidean distances between individuals and the population mean position; max(div) represents the theoretical maximum diversity (diagonal length of the search space); 0.5 is the base probability threshold; and 0.2 is the diversity regulation coefficient and automatically reduces the mixing probability to 0.5 when the population gathers.

#### 3.2.4. Elite Mutation Strategy

Perform directional perturbation on elite individuals according to Equation (31):

(31) $$X_{elite}=X_{elite}+\sigma_{mut}\cdot N(0,1)\;\sigma_{mut}=0.05(ub-lb)(1-t/T_{max})$$

Perform elite pool screening according to Equation (32):

(32) $$N_{elite}=[\eta_{e}\cdot N]\;\eta_{e}=0.15-0.1(t/T_{max})\;ElitePool=\{X(1),\;X(2),\;\ldots ,\;X(N_{elite})\}$$

**Figure 5:**
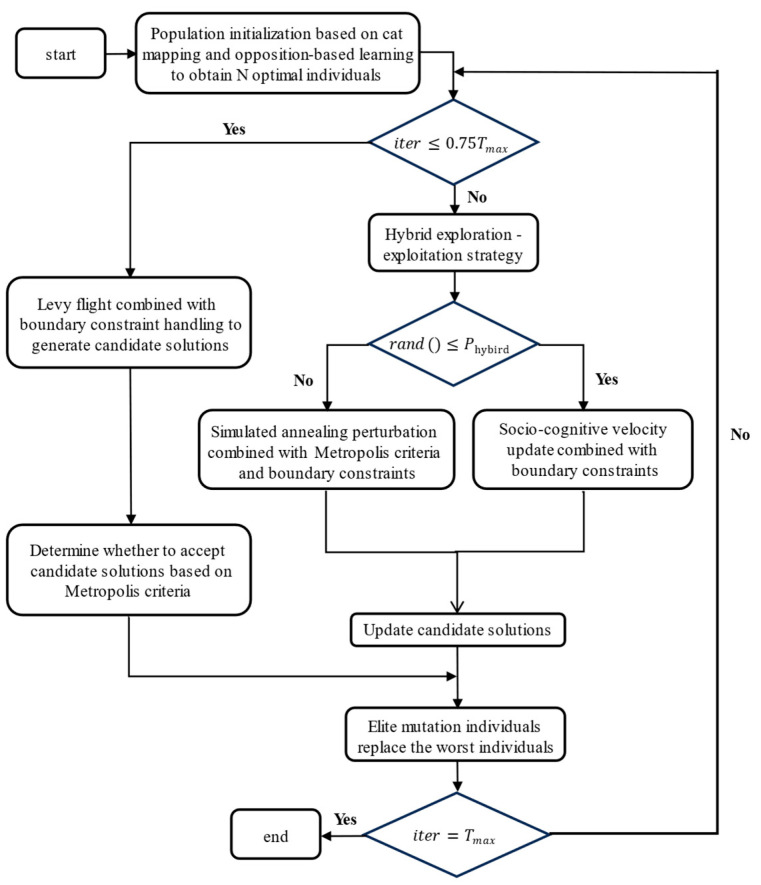
Flowchart of MSCSO algorithm.

Replace the individual with the worst fitness according to Equation (33):

(33) $$X_{\left(N-N_{elite}+1\right)},\ldots ,X_{N}\leftarrow {ElitePool}_{mut}$$

where $X_{elite}$ denotes the elite individuals and $\sigma_{mut}$ denotes the adaptive mutation strength;$N_{elite}$ denotes the number of elite individuals, satisfying 1 ≤ $N_{elite}$ ≤ N; $\eta_{e}$ denotes the elite retention rate, and ${ElitePool}_{mut}$ denotes the new individual set generated by applying mutation operations to the elite pool; and ← denotes the replacement operator, substituting the worst individuals on the left with mutated elites on the right. In summary, the flowchart and pseudocode for MSCSO are shown in Figure 5 and Algorithm 1.
**Algorithm 1:** The MSCSO**Input:** Population size N$,\;\mathrm{Max}\;\mathrm{iterations}\;T_{max}$, Search space [lb, ub] **Output:**$\;\mathrm{Global}\;\mathrm{optimal}\;\mathrm{solution}\;X_{global}$ $\;\mathrm{Initialize}\;\mathrm{the}\;\mathrm{chaotic}\;\mathrm{Cat}\;\mathrm{Map}\;\mathrm{individual}\;X_{cat}$;The opposition-based learning individual $X_{obl}$, the merged population *X*;$\;\mathrm{The}\;\mathrm{Selected}\;\mathrm{Top}\;X_{global}$$\;\mathrm{while}\;t<T_{max}$**do**$\;\;\;\;\;\;\;\;\;\;\;\;\;\;\mathrm{if}\;t<0.75T_{max}$**then**$\;\;\;\;\;\;\;\;\;\;\;\;\;\;\;X_{new}\leftarrow X+0.1\cdot Levy\left(\beta \right)\cdot \left(ub-lb\right)$$\;\;\;\;\;\;\;\;\;\;\;\;\;\;\;\mathrm{Update}\;X\;\mathrm{via}\;\mathrm{Metropolis}\;\mathrm{criterion}\;\mathrm{with}\;T=T_{0}\cdot 0.95^{t}$ 
 
 
 
 
 
**end**$\;\;\;\;\;\;\;\;\;\;\;\;\;\;\mathrm{else}\;\mathrm{if}\;t\geq 0.75T_{max}$**then**$\;\;\;\;\;\;\;\;\;\;\;\;\;\;\;\mathrm{Compute}\;\mathrm{diversity}\;\mathrm{metric}\;div=\frac{1}{N}\sum ∥Xi-\mu \;∥$ 
 
 
 
 
 
 
 
 
 
**for** each individual **do**                  if rand<0.5+0.2(div/max(div))**then**$\;\;\;\;\;\;\;\;\;\;\;\;\;\;\;\;\;\;\;X_{new}\leftarrow X+\delta ,\delta =0.05\left(ub-lb\right)\left(1-t/T_{max}\right)\cdot N\left(0,1\right)$ 
 
 
 
 
 
 
 
 
 
 
 
 
 
 **end**                  else if rand≥0.5+0.2(div/max(div))**then**$\;\;\;\;\;\;\;\;\;\;\;\;\;\;\;\;\;\;\;\mathrm{Update}\;\mathrm{velocity}\;V\leftarrow wV+c_{1}r_{1}\left(X_{global}-X\right)+c_{2}r_{2}\left(\mu -X\right)$$\;\;\;\;\;\;\;\;\;\;\;\;\;\;\;\;\;\;\;X_{new}\leftarrow X+0.1V$** 
 
 
 
 
 
 
 
 
 
 
 
 
 
 end**** 
 
 
 
 
 
 
 
 
end** 
 
 
 
 
 **end**             Perform mutation:$\;X_{mut}\leftarrow X_{elite}+\sigma_{mut}\cdot N\left(0,1\right),\sigma_{mut}=0.05(ub-\;lb(\left(1-t/T_{max}\right)$             Replace worst$\;N_{elite}\;\mathrm{individuals}\;\mathrm{with}\;X_{mut}$$\;\;\;\;\;\;\;\;\;\;\;\;\;\;X_{global}\leftarrow arg\;minf\left(X\right)$              t←t+1**end**

#### 3.2.5. Complexity Analysis

The time complexity of MSCSO is primarily determined by four key parameters: the population size of sand cats (N), the problem dimension (dim), the maximum number of iterations ($T_{max}$), and the evaluation cost per function call (C). Therefore, the time complexity of the MSCSO algorithm is shown in Equation (34).

(34) $$\begin{array}{c} O\left(MSCSO\right)=O\left(\mathrm{parameters}\right)+O\left(\mathrm{initialization}\right)\\ \;\;\;\;\;+O\left(\mathrm{function}\;\mathrm{evaluation}\right)+O(position\;update) \end{array}$$

The specific definitions of each complexity are as follows:

(1) The initialization parameter time is O(1).
(2) Initialization of the population position time O(N×dim), including chaotic mapping and opposition-based learning.
(3) Time required for the global exploration phase $O(0.75T_{max}\times N\times dim)$, involving Lévy flight and Metropolis criterion.
(4) Time required for the local exploitation phase $O(0.25T_{max}\times N\times dim)$, including hybrid exploitation strategy updates.
(5) Time required for elite mutation $O(T_{max}\times N\;log\;N+0.2(T_{max}\times N\times dim))$.
(6) The cost time of the calculation function includes the base evaluation time $O(T_{max}\times N\times C)$, Lévy flight walk time $O(0.75T_{max}\times N\times C)$, and hybrid exploitation strategy evaluation time $O(0.75T_{max}\times N\times C)$, totaling $O(2T_{max}\times N\times C)$.

Therefore, the time complexity of the MSCSO algorithm is as follows:

(35) $$\begin{array}{c} O(MSCSO)=O(1+N\times dim+0.75T_{max}\times N\times dim\\ \;\;+0.25T_{max}\times N\times dim+T_{max}\times N\;log\;N\\ \;\;+0.2T_{max}\times N\times dim+2T_{max}\times N\times C) \end{array}$$

Because $1<<T_{max}\times N\times C$, $1<<T_{max}\times N\times dim$, N log N ≤ N×dim, and $N\times dim\;\leq \;T_{max}\times N\times C$, Equation (35) can be simplified to Equation (36):

(36) $$O(MSCSO)\;\approx \;O(2T_{max}\times N\times C+2.2T_{max}\times N\times dim)$$

When dealing with high-dimensional problems where dim ≈ C, the complexity further reduces to

(37) $$O(MSCSO)\;\approx \;O(4.2T_{max}\times N\times C)$$

## 4. Performance Testing and Analysis of MSCSO

### 4.1. Experimental Environment and Parameter Configuration

#### 4.1.1. Benchmark Function Setup

Benchmark functions can be used to test the optimization ability of algorithms. Before conducting experiments on UAV path planning, the performance of MSCSO needs to be validated on benchmark test functions. The simulation experiments are executed on a 12th Gen Intel Core i7-12700H processor, operating at a base clock speed of 2.30 GHz, equipped with 6 GB of RAM, and running on a Windows 11 64-bit operating system. The computational framework is implemented in MATLAB R2024b. To systematically assess the performance of the MSCSO algorithm, eighteen benchmark functions are selected, with their detailed specifications comprehensively presented in Table 1.

Functions f1–f6 are unimodal benchmark functions, functions f7–f12 are multimodal benchmark functions, and functions f13–f18 are fixed-dimension multimodal benchmark functions that are designed to evaluate the optimization capability of algorithms in low-dimensional complex functions.

#### 4.1.2. Competitive Algorithm Parameter Configuration

To validate the superiority of the MSCSO algorithm, we compare it with seven state-of-the-art optimization algorithms, including novel metaheuristic algorithms such as the original SCSO and Adolescent Identity Search Algorithm (AISA) [29], as well as enhanced variants of classical algorithms: the Grey Wolf Optimizer–particle swarm optimization (HGWOPSO) [30], the Modified Hybrid Seagull Optimization Algorithm (MSSOA) [31], the novel Grey Wolf Optimizer (NGWO) [32], the Chaos-Enhanced Grey Wolf Optimizer with Lévy Flight (CLGWO) [33], and the Chaotic Sand Cat Swarm Optimization (CSCSO) [25]. The experiment is configured with a maximum of 200 iterations and 35 independent runs, with performance metrics including the best, average (Ave), and standard deviation (Std). In addition, when the population size is large, most algorithms can achieve good results, which is not conducive to comparing algorithm performance under limited computing resources. Setting the population size to 35 can help us to fully explore the performance of algorithms under different initial conditions.

#### 4.1.3. Algorithm Comparison

Table 2 systematically compares the competing algorithms through three key metrics: best values, mean values, and standard deviations across benchmark test functions. The best results among the eight algorithms are highlighted in bold. The results from 18 functions demonstrate the superior performance of MSCSO in solution quality and stability. Specifically, MSCSO obtains better optima than comparison algorithms in the majority of functions, though its stochastic solution mechanism leads to marginally inferior results compared to NGWO on specific unimodal functions. Notably, MSCSO maintains significant performance advantages over other algorithms and demonstrates enhanced effectiveness in multimodal function optimization compared to NGWO. These experimental outcomes confirm that the proposed improvements to the original SCSO algorithm strengthen optimization capability, convergence efficiency, and robustness, establishing MSCSO as a competitive methodology among contemporary optimization techniques.

**Figure 6:**
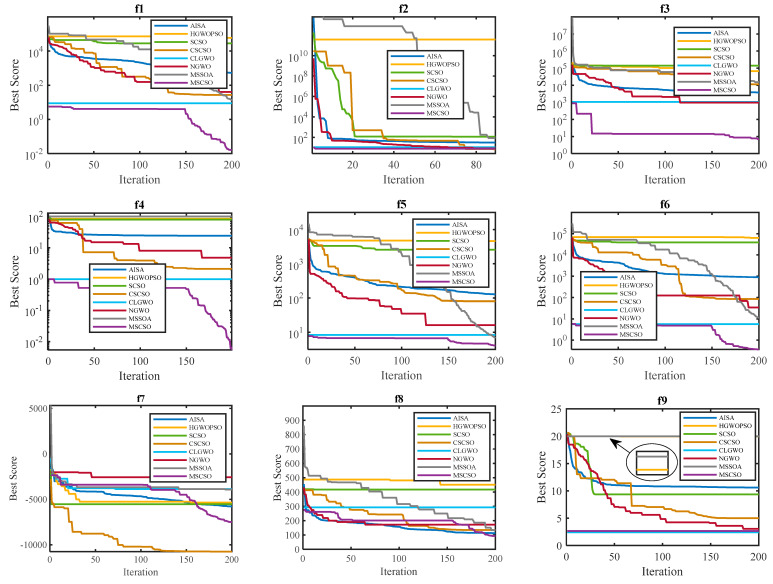

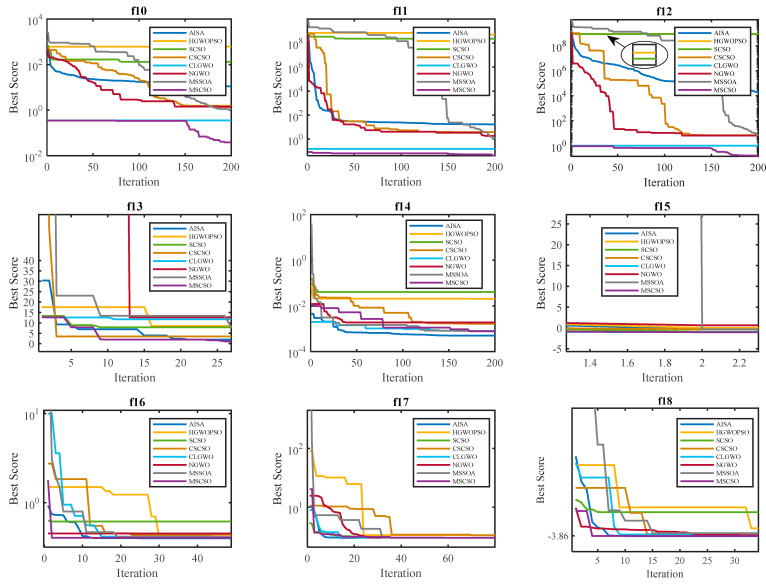
Convergence curves between MSCSO and seven other competitors on 18 benchmark functions.

The convergence behavior of eight optimization algorithms is rigorously evaluated across 18 benchmark functions, as demonstrated in Figure 6. A steeper convergence curve demonstrates an increase in optimization speed, while approaching the *x*-axis reflects an improvement in solution quality. Comparative analysis reveals that the MSCSO algorithm demonstrates superior convergence rates across multiple functional landscapes. Notably, in the f7 test case, MSCSO achieves global optimum identification during preliminary optimization phases, whereas competing methodologies such as HGWOPSO demonstrate protracted convergence trajectories. The results of the fixed-dimension multimodal benchmark functions further confirm MSCSO’s dominance in terminal optimization precision, particularly evident in functions f13, f15, f16, and f18. However, MSCSO’s initial convergence velocity proves suboptimal in unimodal environments such as f9. Comprehensive evaluation across all benchmark functions substantiates MSCSO’s statistically significant superiority in both asymptotic convergence stability and optimization robustness. In addition, most curves of MSCSO will jump out of local optima and continue to converge globally after the 150th iteration, which precisely confirms the effectiveness of the hybrid development strategy.

### 4.2. UAV Path Planning in Complex Environments

We establish a 3D mountainous terrain model (1000 × 800 × 400 m^3^) in MATLAB R2024b for UAV path planning. The model integrates real LiDAR elevation data from Christmas Island, Australia [34]. Negative elevations are truncated to zero for physical consistency, while original values are scaled by 1/5 (model.H=H/5) to meet vertical flight constraints (20–400 m), maintaining realistic geomorphological features and obstacle complexity.

Eight scenarios with varying complexity are generated by configuring obstacle quantities Q (sparse to dense distributions) and waypoint counts (*n* = 6 and *n* = 12), simulating diverse mission requirements. The waypoint configurations correspond to 5-segment and 11-segment flight paths, respectively. In this experiment, the MSCSO algorithm is compared with the original algorithm SCSO. In recent years, the NGWO [32] and CLGWO [33] algorithms have achieved good results in UAV path planning for unmanned aerial vehicles. These two algorithms also demonstrated good optimization results in the experiment in Section 4.1, making them suitable as algorithms for comparison with MSCSO. These algorithms undergo 30 independent trials per scenario (population size 50, maximum iterations 50). Statistical metrics including the mean path length, standard deviation, minimum value, and computational time are recorded to evaluate algorithmic performance disparities.

**Figure 7:**
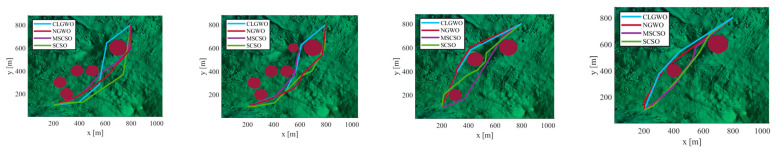

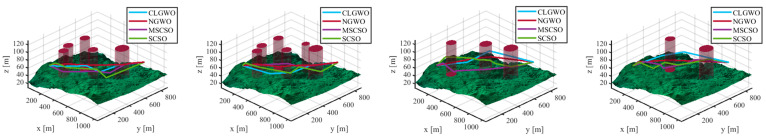
Presentation of the multi-view comparison of path planning under four scenarios (*n* = 6).

**Figure 8:**
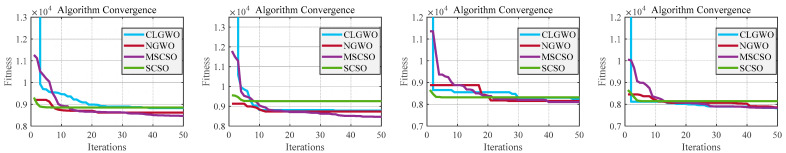
Presentation of the convergence curves of path planning under four scenarios (*n* = 6).

Figure 7 presents a top-view comparison of the path planning results with *n* = 6 waypoints (MSCSO vs. classical algorithms). Although all algorithms can generate feasible paths, significant differences in optimality can occur between different scenarios. Figure 8 demonstrates the evolutionary trajectories of the best fitness values, demonstrating MSCSO’s superior path planning efficiency and accelerated convergence.

Table 3 systematically compares the competing algorithms through three key metrics: best values, mean values, and standard deviations. The best results among the four algorithms are highlighted in bold. As shown in Table 3, MSCSO demonstrates superior path optimization capability and stability in low-dimensional tasks (*n* = 6). In high-obstacle-density scenarios (Q=6), MSCSO achieves a mean path length of 992.86 m, outperforming the suboptimal NGWO algorithm (1012.97 m) by 2.0%, with a minimum value of 980.83 m. These results demonstrate MSCSO’s precision in approaching theoretical optima under an obstacle-dense environment. Furthermore, MSCSO consistently maintains lower path-length standard deviations than competitors. At Q=5, its standard deviation (11.57) reduced by 51.5% compared to CLGWO (23.88), highlighting exceptional stability in solution-space exploration. This stability originates from the algorithm’s adaptive terrain exploration mechanism, which mitigates path oscillations caused by local optima entrapment in conventional methods, thereby producing higher-quality consistent trajectories across diverse scenarios.

**Figure 9:**
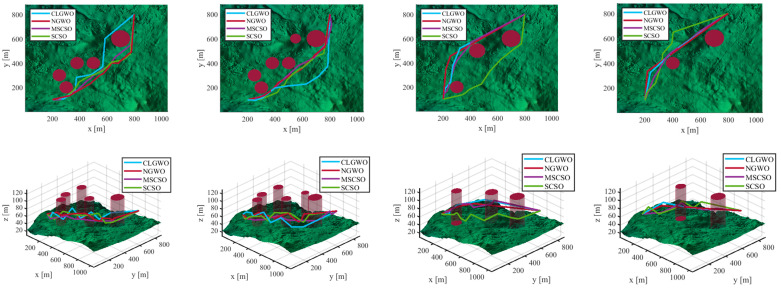
Presentation of the multi-view comparison of path planning under four scenarios (*n* = 12).

**Figure 10:**
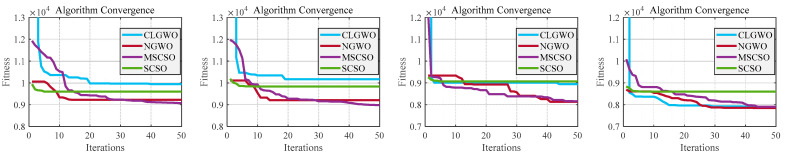
Presentation of the convergence curves of path planning under four scenarios (*n* = 12).

Figure 9 displays a top-view comparison of path generation with *n* = 12 waypoints between MSCSO and the state-of-the-art algorithms. Although all methods have generated feasible paths as shown in Figure 10, there are differences between the paths. Table 4 demonstrates MSCSO’s superiority when the number of waypoints increases to *n* = 12 (11 flight segments). The best results among the four algorithms are highlighted in bold. Despite the increased complexity of the task, the average path length of MSCSO only increased by 1.0% (from 981.10 m to 990.69 m), while SCSO increased by 7% (from 1022.32 m to 1094.27 m). This demonstrates that MSCSO has reduced sensitivity to dimension expansion, ensuring the reliability of high-dimensional complex missions. The experimental results conclusively demonstrate that MSCSO achieves superior improvements in optimization accuracy, stability, and scalability across low- and high-dimensional missions. The algorithm demonstrates robust adaptability to diverse complex scenarios, delivering an efficient and reliable solution for autonomous UAV navigation in real-world terrains.

## 5. Conclusions and Future Perspectives

This study proposes a multi-strategy enhanced Sand Cat Swarm Optimization (MSCSO) algorithm to address critical challenges in three-dimensional UAV path planning within complex low-altitude environments. The algorithm achieves performance breakthroughs through four innovative mechanisms: (1) A population initialization strategy based on chaotic mapping and opposition-based learning, which generates high-dimensional chaotic sequences using Cat mapping and enhances spatial coverage of initial solutions via Top−N selection. (2) A Lévy flight–Metropolis hybrid exploration mechanism, coupling long-range jumps with dynamic solution acceptance strategies during early iterations to significantly improve global search capabilities. (3) A simulated annealing–particle swarm hybrid exploitation mechanism, balancing local convergence accuracy and computational efficiency in mid-to-late iterations through nonlinear decaying inertia weights and dynamic cognitive coefficients. (4) A dynamic elite mutation strategy employing adaptive Gaussian perturbations to escape local optima. Experimental validation demonstrates that MSCSO improves convergence speed by 23% and reduces standard deviation by 18–35% compared to the original SCSO across 18 benchmark functions, particularly excelling in global optimization for multimodal functions f9–f12. For three-dimensional mountainous environments with kinematic constraints, the algorithm shortens the path lengths by an average of 15.7% in 6- and 12-waypoint tasks, stabilizes computation time within 18–25 s, and ensures the physical feasibility of flight control through a path smoothness cost function, offering reliable solutions for low-altitude economy applications such as logistics and disaster relief.

Future research should focus on the following: (1) Developing a real-time trajectory replanning frameworks for dynamic obstacle environments, integrating sensor data fusion and online learning mechanisms to address sudden threats. (2) Establishing multi-UAV collaborative planning systems that resolve distributed optimization and conflict mitigation under communication latency constraints. (3) Validating algorithmic robustness against practical disturbances like inertial navigation errors and actuator response delays through hardware-in-the-loop simulations. (4) Comparative studies with other chaotic maps under the NIST SP800-22 standards [35]. Additionally, quantum computing-based variants (e.g., qubit encoding and quantum gate operations) can be explored to enhance computational scalability in ultra-large-scale urban airspace networks (>100 UAVs). These extensions will accelerate the translation of theoretical advantages into engineering practicality, providing core support for intelligent low-altitude economy infrastructure.

## Figures and Tables

**Table 1:** Benchmark functions.

Type	ID	Description	Range	$f_{min}$
Unimodal Test Functions	f1	Sphere Function	[−100, 100]	0
	f2	Schwefel’s Problem 2.22	[−10, 10]	0
	f3	Schwefel’s Problem 1.2	[−100, 100]	0
	f4	Schwefel’s Problem 2.21	[−100, 100]	0
	f5	Generalized Rosenbrock’s Function	[−30, 30]	0
	f6	Step Function	[−100, 100]	0
Multimodal Test Functions	f7	Generalized Schwefel’s Problem 2.26	[−500, 500]	−12,569.487
	f8	Generalized Rastrigin’s Function	[−5.12, 5.12]	0
	f9	Ackley’s Function	[−32, 32]	0
	f10	Generalized Griewank’s Function	[−600, 600]	0
	f11	Generalized Penalized Function	[−50, 50]	0
	f12	Generalized Penalized Functions	[−50, 50]	0
Fixed-Dimensional Multimodal Test Functions	f13	Shekel’s Foxholes Function	[−65.536, 65.536]	~0.998
	f14	Kowalik’s Function	[−5, 5]	~0.0003
	f15	Six-Hump Camel Back Function	[−5, 5]	−1.0316
	f16	Branin Function	[−5, 0] to [10, 15]	0.3979
	f17	Goldstein–Price Function	[−2, 2]	3
	f18	Hartman’s Function	[0, 1]	−3.86

**Table 2:** The experimental results are compared on 18 benchmark functions.

ID	Metrics	AISA	HGWOPSO	SCSO	CSCSO	MSCSO	CLGWO	NGWO	MSSOA
f1	Best	2.446 × 10^2^	4.743 × 10^4^	1.120 × 10^4^	5.920 × 10^0^	**2.479 × 10^−5^**	5.734 × 10^0^	2.066 × 10^1^	1.441 × 10^0^
	Ave	7.812 × 10^2^	5.986 × 10^4^	3.841 × 10^4^	1.192 × 10^2^	**4.424 × 10^−1^**	8.381 × 10^0^	6.958 × 10^1^	7.645 × 10^0^
	Std	5.489 × 10^2^	6.109 × 10^3^	1.705 × 10^4^	9.231 × 10^1^	**6.939 × 10^−1^**	1.252 × 10^0^	3.871 × 10^1^	6.019 × 10^0^
f2	Best	9.057 × 10^0^	1.748 × 10^7^	2.755 × 10^1^	7.443 × 10^−1^	**1.879 × 10^−1^**	9.685 × 10^0^	1.539 × 10^0^	2.866 × 10^−1^
	Ave	1.723 × 10^1^	5.940 × 10^10^	7.391 × 10^8^	4.660 × 10^0^	4.039 × 100	1.154 × 10^1^	2.860 × 10^0^	**6.318 × 10^−1^**
	Std	4.819 × 10^0^	1.373 × 10^11^	4.048 × 10^9^	3.097 × 10^0^	7.276 × 10^−1^	7.335 × 10^−1^	6.607 × 10^−1^	**2.632 × 10^−1^**
f3	Best	1.445 × 10^3^	6.301 × 10^4^	2.593 × 10^4^	2.138 × 10^2^	**2.011 × 10^−1^**	1.944 × 10^2^	1.297 × 10^2^	5.539 × 10^3^
	Ave	3.505 × 10^3^	7.568 × 10^4^	9.875 × 10^4^	1.122 × 10^4^	**1.585 × 10^0^**	9.366 × 10^2^	5.222 × 10^2^	1.192 × 10^4^
	Std	1.242 × 10^3^	8.091 × 10^3^	3.587 × 10^4^	9.796 × 10^3^	**1.791 × 10^0^**	2.854 × 10^2^	3.272 × 10^2^	4.015 × 10^3^
f4	Best	1.492 × 10^1^	7.664 × 10^1^	3.893 × 10^1^	8.477 × 10^−1^	**2.966 × 10^−2^**	9.833 × 10^−1^	1.889 × 10^0^	8.858 × 10^0^
	Ave	2.351 × 10^1^	8.680 × 10^1^	7.197 × 10^1^	4.715 × 10^0^	**4.675 × 10^−1^**	9.869 × 10^−1^	4.684 × 10^0^	5.351 × 10^1^
	Std	4.442 × 10^0^	3.409 × 10^0^	1.429 × 10^1^	3.026 × 10^0^	**4.316 × 10^−3^**	5.280 × 10^−3^	1.301 × 10^0^	3.539 × 10^1^
f5	Best	9.964 × 10^3^	9.663 × 10^7^	4.450 × 10^5^	**6.374 × 10^1^**	9.048 × 10^1^	4.640 × 10^2^	2.126 × 10^2^	8.156 × 10^1^
	Ave	1.096 × 10^5^	2.039 × 10^8^	8.274 × 10^7^	3.029 × 10^3^	**2.122 × 10^2^**	6.030 × 10^2^	1.544 × 10^3^	1.019 × 10^3^
	Std	1.583 × 10^5^	5.298 × 10^7^	6.811 × 10^7^	5.161 × 10^3^	**5.888 × 10^1^**	6.863 × 10^1^	1.187 × 10^3^	1.180 × 10^3^
f6	Best	2.297 × 10^2^	4.738 × 10^4^	1.304 × 10^4^	3.512 × 10^0^	**1.763 × 10^−1^**	5.321 × 10^0^	6.779 × 10^0^	7.835 × 10^−1^
	Ave	7.886 × 10^2^	6.137 × 10^4^	3.843 × 10^4^	1.196 × 10^2^	**5.094 × 10^−1^**	5.729 × 10^0^	7.224 × 10^0^	1.016 × 10^1^
	Std	4.936 × 10^2^	5.881 × 10^3^	1.547 × 10^4^	1.166 × 10^2^	**1.595 × 10^−1^**	1.657 × 10^−1^	2.446 × 10^−1^	7.219 × 10^0^
f7	Best	−6.730 × 10^3^	−5.748 × 10^3^	−7.361 × 10^3^	**−1.257 × 10^4^**	−6.848 × 10^3^	−4.205 × 10^3^	−4.072 × 10^3^	−4.430 × 10^3^
	Ave	−6.153 × 10^3^	−5.517 × 10^3^	−6.302 × 10^3^	**−1.257 × 10^4^**	−6.154 × 10^3^	−3.803 × 10^3^	−3.863 × 10^3^	−4.044 × 10^3^
	Std	5.214 × 10^2^	2.025 × 10^2^	9.756 × 10^2^	**3.013 × 10^−1^**	6.726 × 10^2^	4.248 × 10^2^	2.036 × 10^2^	3.437 × 10^2^
f8	Best	9.000 × 10^1^	3.936 × 10^2^	2.768 × 10^2^	**1.552 × 10^0^**	1.116 × 10^2^	3.045 × 10^2^	4.186 × 10^1^	1.533 × 10^2^
	Ave	1.076 × 10^2^	4.128 × 10^2^	3.234 × 10^2^	**3.474 × 10^1^**	1.239 × 10^2^	3.155 × 10^2^	9.861 × 10^1^	1.836 × 10^2^
	Std	2.035 × 10^1^	2.108 × 10^1^	7.816 × 10^1^	5.392 × 10^1^	1.145 × 10^1^	**9.834 × 10^0^**	3.873 × 10^1^	4.633 × 10^1^
f9	Best	9.924 × 10^0^	1.996 × 10^1^	5.749 × 10^0^	**1.333 × 10^0^**	2.269 × 10^0^	2.282 × 10^0^	2.055 × 10^0^	1.999 × 10^1^
	Ave	1.170 × 10^1^	1.997 × 10^1^	1.898 × 10^1^	3.622 × 10^0^	2.692 × 10^0^	**2.546 × 10^0^**	2.969 × 10^0^	1.999 × 10^1^
	Std	1.060 × 10^0^	4.694 × 10^−3^	3.378 × 10^0^	1.330 × 10^0^	1.741 × 10^−1^	1.816 × 10^−1^	5.212 × 10^−1^	**0.000 × 10^0^**
f10	Best	9.298 × 10^0^	5.722 × 10^2^	1.331 × 10^2^	1.119 × 10^0^	**3.812 × 10^−2^**	1.868 × 10^−1^	1.376 × 10^0^	1.035 × 10^0^
	Ave	1.144 × 10^1^	5.874 × 10^2^	2.432 × 10^2^	1.513 × 10^0^	**4.809 × 10^−2^**	2.829 × 10^−1^	1.606 × 10^0^	1.048 × 10^0^
	Std	2.429 × 10^0^	1.798 × 10^1^	1.411 × 10^2^	3.751 × 10^−1^	**8.675 × 10^−3^**	8.678 × 10^−2^	2.034 × 10^−1^	1.494 × 10^−2^
f11	Best	8.566 × 10^0^	1.708 × 10^8^	2.438 × 10^5^	**1.062 × 10^−2^**	1.257 × 10^−2^	5.493 × 10^−2^	8.058 × 10^−1^	2.346 × 10^−1^
	Ave	3.040 × 10^1^	4.301 × 10^8^	1.983 × 10^8^	1.232 × 10^0^	**2.462 × 10^−2^**	1.350 × 10^−1^	1.871 × 10^0^	1.910 × 10^0^
	Std	1.936 × 10^1^	1.252 × 10^8^	1.815 × 10^8^	1.563 × 10^0^	**9.381 × 10^−3^**	3.492 × 10^−2^	5.440 × 10^−1^	1.455 × 10^0^
f12	Best	7.840 × 10^1^	5.577 × 10^8^	9.697 × 10^6^	2.509 × 10^−1^	**7.872 × 10^−2^**	8.217 × 10^−1^	3.310 × 10^0^	1.544 × 10^0^
	Ave	1.291 × 10^4^	9.534 × 10^8^	4.201 × 10^8^	5.589 × 10^0^	**2.102 × 10^−1^**	1.041 × 10^0^	5.596 × 10^0^	4.911 × 10^0^
	Std	3.256 × 10^4^	1.718 × 10^8^	3.803 × 10^8^	4.973 × 10^0^	**1.031 × 10^−1^**	1.409 × 10^−1^	1.202 × 10^0^	2.438 × 10^0^
f13	Best	**9.980 × 10^−1^**	1.002 × 10^0^	1.992 × 10^0^	1.992 × 10^0^	**9.980 × 10^−1^**	**9.980 × 10^−1^**	2.272 × 10^0^	**9.980 × 10^−1^**
	Ave	1.329 × 10^0^	1.263 × 10^0^	1.030 × 10^1^	2.651 × 10^0^	**9.980 × 10^−1^**	**9.980 × 10^−1^**	3.198 × 10^0^	**9.980 × 10^−1^**
	Std	5.739 × 10^−1^	4.468 × 10^−1^	9.740 × 10^0^	1.141 × 10^0^	**2.898 × 10^−10^**	4.161 × 10^−10^	1.050 × 10^0^	4.881 × 10^−7^
f14	Best	**3.075 × 10^−4^**	1.037 × 10^−3^	2.255 × 10^−3^	5.752 × 10^−4^	5.834 × 10^−4^	3.835 × 10^−4^	3.100 × 10^−4^	5.258 × 10^−4^
	Ave	1.519 × 10^−3^	5.927 × 10^−3^	4.266 × 10^−2^	2.658 × 10^−3^	1.253 × 10^−3^	**7.871 × 10** ^−4^	9.605 × 10^−4^	1.433 × 10^−3^
	Std	3.987 × 10^−3^	8.126 × 10^−3^	3.951 × 10^−2^	3.935 × 10^−3^	4.392 × 10^−4^	**2.149 × 10** ^−4^	2.770 × 10^−3^	3.579 × 10^−3^
f15	Best	−1.032 × 10^0^	−1.032 × 10^0^	−1.032 × 10^0^	−1.032 × 10^0^	**−1.032 × 10^0^**	−1.032 × 10^0^	−1.032 × 10^0^	−1.032 × 10^0^
	Ave	−1.032 × 10^0^	−1.029 × 10^0^	−7.888 × 10^−1^	−1.032 × 10^0^	**−1.032 × 10^0^**	−1.031 × 10^0^	−1.032 × 10^0^	−1.032 × 10^0^
	Std	5.952 × 10^−6^	3.013 × 10^−3^	3.456 × 10^−1^	1.479 × 10^−4^	**3.643 × 10^−6^**	7.708 × 10^−6^	1.939 × 10^−5^	1.147 × 10^−4^
f16	Best	3.979 × 10−^1^	3.981 × 10^−1^	3.979 × 10^−1^	3.979 × 10^−1^	**3.979 × 10^−1^**	3.979 × 10^−1^	3.979 × 10^−1^	3.979 × 10^−1^
	Ave	3.981 × 10^−1^	4.036 × 10^−1^	1.038 × 10^0^	3.979 × 10^−1^	**3.979 × 10^−1^**	3.980 × 10^−1^	4.004 × 10^−1^	7.076 × 10^−1^
	Std	**3.067 × 10^−7^**	8.358 × 10^−3^	1.233 × 10^0^	1.190 × 10^−5^	8.553 × 10^−6^	6.955 × 10^−4^	3.070 × 10^−3^	1.178 × 10^0^
f17	Best	**3.000 × 10^0^**	3.001 × 10^0^	**3.000 × 10^0^**	**3.000 × 10^0^**	**3.000 × 10^0^**	**3.000 × 10^0^**	**3.00^0^ × 10^0^**	**3.000 × 10^0^**
	Ave	**3.000 × 10^0^**	3.029 × 10^0^	2.359 × 10^1^	**3.000 × 10^0^**	**3.000 × 10^0^**	**3.000 × 10^0^**	**3.00^0^ × 10^0^**	**3.000 × 10^0^**
	Std	1.046 × 10^−4^	3.121 × 10^−2^	3.046 × 10^1^	2.079 × 10^−5^	4.151 × 10^−5^	**6.396 × 10^−6^**	2.322 × 10^−4^	1.676 × 10^−4^
f18	Best	−3.863 × 10^0^	−3.859 × 10^0^	−3.858 × 10^0^	−3.863 × 10^0^	**−3.863 × 10^0^**	−3.863 × 10^0^	−3.862 × 10^0^	−3.863 × 10^0^
	Ave	−3.863 × 10^0^	−3.854 × 10^0^	−3.627 × 10^0^	−3.855 × 10^0^	**−3.863 × 10^0^**	−3.861 × 10^0^	−3.860 × 10^0^	−3.859 × 10^0^
	Std	1.937 × 10^−5^	2.516 × 10^−3^	3.342 × 10^−1^	2.180 × 10^−2^	**1.450 × 10^−5^**	3.538 × 10^−3^	1.897 × 10^−3^	3.671 × 10^−3^

**Table 3:** Complex environment simulation experiment data (*n* = 6).

Obstacle Count Q	Algorithm	Ave	Std	Best	Running Time (s)
2	CLGWO	956.93	**6.07**	954.25	0.10
	NGWO	969.06	12.32	961.25	**0.08**
	MSCSO	**942.01**	8.77	**931.14**	0.23
	SCSO	983.53	23.67	975.49	0.09
3	CLGWO	978.07	16.62	965.58	0.11
	NGWO	1002.11	12.89	980.15	0.10
	MSCSO	**959.91**	**5.23**	**944.05**	0.27
	SCSO	1019	27.12	987.70	**0.09**
5	CLGWO	1016.89	23.88	1000.43	0.21
	NGWO	1000.15	21.01	977.31	**0.15**
	MSCSO	**981.10**	**11.57**	**945.77**	0.38
	SCSO	1022.32	43.07	1009.79	0.15
6	CLGWO	1041.31	42.44	1015.58	0.14
	NGWO	1012.97	11.00	998.70	**0.13**
	MSCSO	**992.86**	**4.21**	**980.83**	0.39
	SCSO	1053.55	65.66	1021.18	**0.13**

**Table 4:** Complex environment simulation experiment data (*n* = 12).

Obstacle Count Q	Algorithm	Ave	Std	Best	Running Time (s)
2	CLGWO	968.83	10.44	960.14	0.16
	NGWO	977.08	7.37	970.18	**0.13**
	MSCSO	**946.81**	**6.92**	**937.93**	0.35
	SCSO	1054.59	31.44	1024.35	**0.13**
3	CLGWO	997.07	16.34	986.15	0.17
	NGWO	1014.31	10.03	1005.26	0.15
	MSCSO	**964.10**	**3.76**	**949.85**	0.41
	SCSO	1060.46	51.22	1028.37	**0.14**
5	CLGWO	1019.95	30.79	1002.72	0.22
	NGWO	1009.95	22.17	983.77	0.19
	MSCSO	**990.69**	**16.69**	**963.94**	0.51
	SCSO	1094.27	45.33	1050.72	**0.17**
6	CLGWO	1072.71	40.01	1049.11	0.23
	NGWO	1023.23	12.11	1014.84	0.20
	MSCSO	**1000.88**	**5.97**	**965.69**	0.52
	SCSO	1103.77	30.62	1050.51	**0.19**

## Data Availability

All data are contained within the article.
